# Droplet digital PCR-based pathogen monitoring system improves identifying *Haemaphysalis longicornis* infected with *Bandavirus dabieense* and other tick-borne pathogens at low concentrations

**DOI:** 10.1186/s13071-026-07548-1

**Published:** 2026-07-01

**Authors:** Yujin Baek, Jae-Hun Jeong, Da-Eun Jeong, Jun-Gu Kang, Yong Shin, Il-Hwan Kim

**Affiliations:** 1 Division of Biomedical Metrology, Korea Research Institute of Standards and Science, Daejeon, Republic of Korea; 2 Department of Biotechnology, College of Life Science and Biotechnology, Yonsei University, Seoul, Republic of Korea; 3 Department of Precision Engineering Science, University of Science and Technology, Daejeon, Republic of Korea; 4 Korea Zoonosis Research Institute, Jeonbuk National University, Iksan, Republic of Korea; 5 Department of Precision Measurement Science, University of Science and Technology, Daejeon, Republic of Korea

**Keywords:** Droplet digital PCR, *Haemaphysalis longicornis*, *Bandavirus dabieense*, Tick-borne pathogens

## Abstract

**Background:**

Asian long-horned ticks, *Haemaphysalis longicornis,* vector viruses, bacteria and parasites that cause major zoonotic diseases in human. In order to prevent and manage the spread of tick-borne zoonosis, it is important to monitor pathogen prevalence in wild tick populations using molecular diagnostics with high detection performance. In this study, we introduce a novel droplet digital PCR (ddPCR)-based pathogen monitoring system to identify *Haemaphysalis longicornis* ticks infected with *Bandavirus dabieense* and other zoonotic pathogens at low concentrations with higher sensitivity and accuracy from existing molecular detection methods.

**Methods:**

Using metrologically certified *B. dabieense* RNA reference materials, we directly compared the detection limits between ddPCR and quantitative PCR (qPCR). Then, among 502 individual *H. longicornis* adult ticks collected from fields, we determined the detection rate of ticks infected with *B. dabieense*. We also investigated tick innate immune system, JAK/STAT, for its relevance in identifying ticks infected with low concentrations of *B. dabieense.* From *B. dabieense*-infected ticks, we examined for other tick-borne pathogens and nucleic acid concentrations of identified pathogens using ddPCR*.*

**Results:**

Our ddPCR-based method showed high sensitivity by 20 times lower detection limits than that of qPCR. Detection rate of ticks infected with *B. dabieense* from fields was 35.9%. Among ticks infected with low *B. dabieense* virus concentrations, defined by the *B. dabieense* RNA copy numbers in bottom 25% quartiles observed from infected ticks, STAT expression level was significantly higher compared to laboratory strains and uninfected field populations. Lastly, multiplexed ddPCR results indicated that individual ticks can harbor up to four different pathogens concurrently.

**Conclusions:**

Our newly developed ddPCR-based pathogen monitoring system improves the identification of *H. longicornis* infected with *B. dabieense* and other tick-borne pathogens at low nucleic acid concentrations compared with qPCR. In addition, our results suggest that STAT expression may provide exploratory and complementary insight into tick innate immune activation in low-copy *B. dabieense* infections, and that ddPCR-based multiplexing can detect co-infections with multiple tick-borne pathogens in individual ticks. These findings highlight the potential of ddPCR as a sensitive and practical tool for surveillance and management of tick-borne zoonotic pathogens in vector populations.

**Graphical Abstract:**

**Figure:**
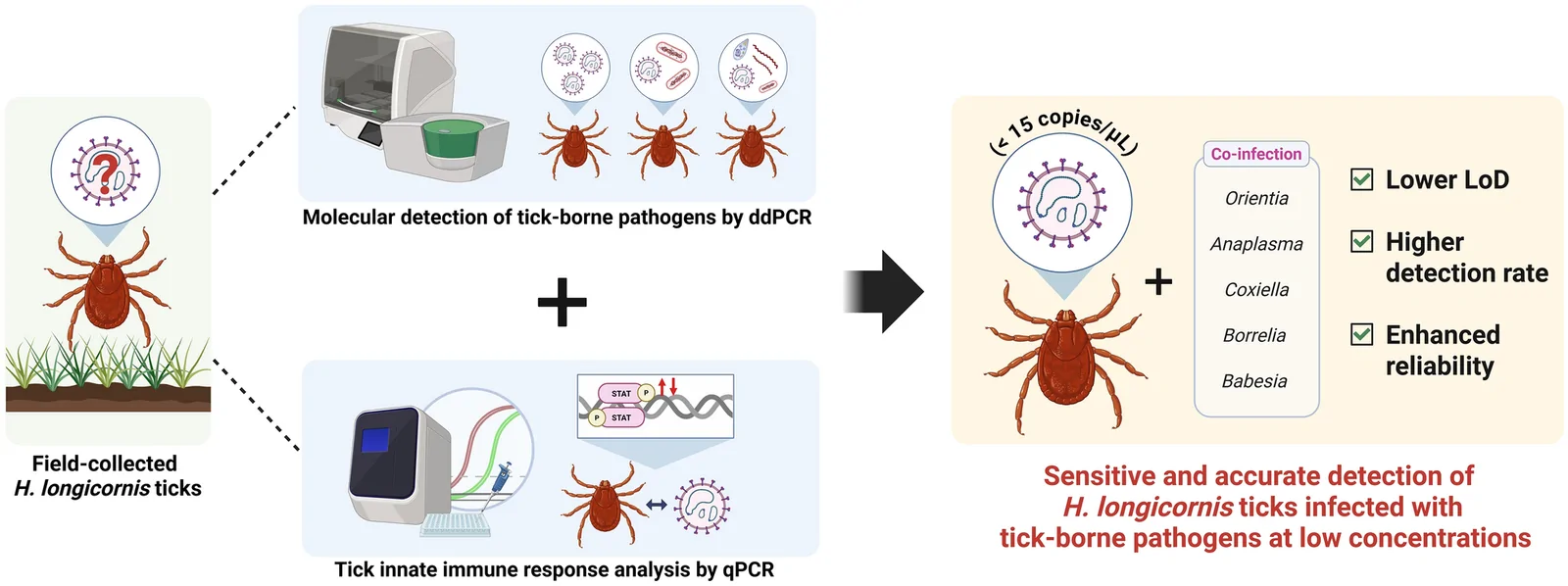

**Supplementary Information:**

The online version contains supplementary material available at https://doi.org/10.1186/s13071-026-07548-1.

## Background

*Haemaphysalis longicornis* is an ixodid tick species widely distributed in East Asia and the main vector species for transmitting *Bandavirus dabieense* virus into human which is a causative agent of Severe Fever with Thrombocytopenia Syndrome (SFTS) [1, 2]. *H. longicornis* is also known to harbor bacteria and parasites including *Anaplasma*, *Babesia*, *Borrelia*, *Coxiella* and *Rickettsia* species which cause various tick-borne zoonosis in human [3, 4]. Therefore, surveillance of field-collected *H. longicornis* populations for the presence of tick-borne pathogens is important to assess the risk of disease transmission from ticks and establish effective prevention strategies [5].

Nucleic acid amplification test-based molecular diagnostic technologies, including quantitative PCR (qPCR), are widely used for pathogen surveillance from *H. longicornis* captured in nature. Current surveillance of *B. dabieense* in *H. longicornis* relies on qPCR-based methods but detection rates based on these methods range only from 0.48 to 12.6% [6–9]. The low detection rate is potentially influenced by the limited sensitivity of qPCR targeting low-abundance nucleic acids from complex environmental samples [10, 11]. Therefore, molecular diagnostics with higher detection performance is necessary to improve the surveillance capability for accurately identifying ticks infected with low pathogen concentrations.

Droplet digital PCR (ddPCR) is a next-generation molecular diagnostic technology that directly quantitate nucleic acid molecules by partitioning the reaction mixture into several thousands of nanoliter-sized droplets and amplify target molecules independently within each droplet [12]. Thus, ddPCR allows for improved detection sensitivity, accuracy as well as direct quantitation of nucleic acids even at low concentrations compare to qPCR [13, 14]. Despite these technical advantages, only a few studies have investigated the potential use of ddPCR technology as a pathogen surveillance system for ticks [15]. Therefore, it is necessary to investigate whether ddPCR-based monitoring system has a potential implication for detecting pathogens with high sensitivity and specificity from total nucleic acids isolated from field-collected *H. longicornis*. Moreover, since ddPCR is capable of multiplexed detection of target nucleic acids, it is possible to examine the pathogen diversity and frequency within the individual *H. longicornis* in nature.

To address the limitation of current PCR-based pathogen surveillance methods for disease transmitting arthropod vectors, we developed a ddPCR-based pathogen monitoring system for detecting *B. dabieense* and other tick-borne pathogens in *H. longicornis* even at low concentrations of nucleic acid. In addition, we performed a brief exploratory analysis of tick innate immune responses to assess whether STAT expression might provide complementary information in ticks with low-copy infections. We also applied ddPCR multiplexing to investigate co-infections with multiple tick-borne pathogens in individual ticks.

## Methods

### Tick collection

Adult *H. longicornis* ticks used for pathogen detection in this study were collected using flags in Jeju Island, Republic of Korea. Collected tick samples were identified to species and developmental stages through microscopic examination. After identification processes, ticks were stored at − 80 °C. Laboratory‑reared adult *H. longicornis* ticks were used for the analysis of STAT gene expression. This colony was originally established from ticks collected from the bushes of a peach orchard in Sangju, Republic of Korea using CO_2_-baited traps, and has since been continuously maintained in the Laboratory of Vector Biology at Kyungpook National University. All procedures for rearing *H. longicornis* tick were conducted in accordance with the Animal Protection Act of the Republic of Korea and were approved by the Animal Ethics Committee of Kyungpook National University (protocol number: 2023‑0045).

### Nucleic acid extraction and cDNA synthesis

*H. longicornis* were homogenized individually, and then total DNA and RNA was extracted separately per individual tick homogenates using the Clear-S™ DNA/RNA Extraction Kit (InVirusTech, South Korea). The extracted RNA was synthesized into cDNA using a SuperScript™ IV VILO™ Master Mix Kit with ezDNase (ThermoFisher Scientific, South Korea). Prepared DNA and cDNA samples were stored at −80 °C until further usage.

### Reference nucleic acid preparation

Reference nucleic acids for assay validation were prepared as follows. *B. dabieense* RNA used in this study was obtained from certified RNA reference materials consisting of synthetic *B. dabieense* genomic RNA with assigned copy numbers, as previously described [16]. Genomic DNA of *Orientia tsutsugamushi* and *Anaplasma phagocytophilum* were extracted from cultured bacteria. Synthetic plasmid DNAs containing the target regions of *Coxiella burnetii* IS1111, *Borrelia afzelii* 16S rRNA and *Babesia microti* 18S rRNA were used as reference nucleic acids for assay validation.

### Design and validation of in-house primers and probes

Pathogen-specific universal primers and dual-labeled hydrolysis probes were designed to target *B. dabieens*e, *O. tsutsugamushi*, *A. phagocytophilum*, *C. burnetii*, *Borrelia* spp. and *Babesia* spp. These primer–probe sets were designed to amplify gene regions among representative genotypes, strains or species of each target pathogen (Additional file 1: Table S1). In-house designed primer and probe sets were validated using reference nucleic acids. The linearity and detection limit for these assays were evaluated using tenfold serial dilutions of the corresponding template. The specificity of each assay was evaluated by testing each primer–probe set against non-targeted reference nucleic acids to confirm the absence of cross-reaction amplification. Annealing temperatures between 58 and 60 °C, amplification cycle numbers between 40 and 45, and several primer–probe working concentrations were evaluated using serially diluted RNA samples from the *B. dabieense* RNA reference material. After identifying optimal reaction conditions, ddPCR and qPCR analyses were performed at an annealing temperature of 58 °C with 40 amplification cycles.

### Droplet digital PCR experiments

Reactions were prepared using ddPCR Supermix for Probes (No dUTP) (Bio-Rad Laboratories, USA) with final concentrations of 303 nM of each primer and 152 nM of each probe, according to the manufacturer’s instructions. Droplets were generated using an Automated Droplet Generator (Bio-Rad Laboratories, USA). After PCR amplification, droplets were analyzed using a QX200 Droplet Reader (Bio-Rad Laboratories, USA) and QuantaSoft software version 1.7.4 (Bio-Rad Laboratories, USA). All thresholds were manually set to distinguish between positive and negative droplets. *H. longicornis* cDNA was used to detect *B. dabieense*, and total DNA isolated from *H. longicornis* was used to detect *O. tsutsugamushi*, *A. phagocytophilum*, *C. burnetii*, *Borrelia* spp. and *Babesia* spp. Duplex ddPCR assays were set up for multiplexed detection within each reaction well. FAM channel of the QX200™ reader was assigned for the detection of *B. dabieense*, *C. burnetii* and *Borrelia* spp (Table 1). HEX channel was assigned for detection of *O. tsutsugamushi*, *A. phagocytophilum* and *Babesia* spp (Table 1).

**Table 1:** Primer and probe sequences used in this study

Species	Target gene	Sequences (5′–3′)
*Bandavirus dabieense*	RdRp	F: TTCCACAGGCACTTGGTTAG
		P: FAM-TGGAAGGAGGGGCCTCATTCTCTCT-BHQ1
		R: CCCCATGAAGGTTCCAAACA
*Orientia tsutsugamushi*	56-kDa	F: GGTATGACAATTGCTCCAGG
		P: HEX-AGAGCAGAGCTAGGGGTTATGTACCT-BHQ1
		R: CACGATCCGCTATACTTATAGG
*Anaplasma phagocytophilum*	ankA	F: CTGTATGCTACTGTGGGAGC
		P: HEX-GCGCAAGCATCAGATGCGGCGTCAT-BHQ1
		R: TCCAGAAGATGTTCGTTCCG
*Coxiella burnetii*	IS1111	F: ATCGCGTATCTTTAACAGCGC
		P: FAM-CACCGTTTTACCCCGCACAAACCGC-BHQ1
		R: GAGCGAACCATTGGTATCGG
*Borrelia* spp.	16 s rRNA	F: ATAAGTAACCGGCCTGAGAGG
		P: FAM-AATGGGCGAAAGCCTGACGGAGCG-BHQ1
		R: ACGTAATTAGCCGGGGCTTAT
*Babesia* spp.	18 s rRNA	F: CCGTCGTAGTCCTAACCATAAAC
		P: HEX-GACTCCTTCAGCACCTTGAGAGA-BHQ1
		R: TTCAGCCTTGCGACCATAC
*Haemaphysalis longicornis*	β-actin	F: TCCTCACTCTGAAGTACCCC
		R: TCTCGAACATGATCTGCGTC
	STAT	F: GTCACCAGCACCTTCATCATC
		R: CGCTCTTCAGCAAGGAGTTT

### Quantitative PCR experiments

Reactions were performed using RbTaq™ Multi‑Probe qPCR PreMIX (Enzynomics, South Korea) according to the manufacturer’s protocol. Reaction mixtures contained 333 nM of each primer 167 nM of each probe. Cycle threshold (Ct) values higher than 39 were considered negative. STAT gene from *H. longicornis* genome was used for the analysis of innate immune gene expression. qPCR was performed as described previously. The reaction mixtures contained cDNA from individual *H. longicornis*, 500 nM of forward and reverse primers (Table 1). Results were calculated as ΔCt values where ΔCt is the difference between the cycle threshold value of the target gene and the Ct value of the *H. longicornis* housekeeping gene, β-actin.

### Statistical analysis

Statistical analyses were performed using GraphPad Prism 10.0 (GraphPad Software, USA). Comparisons of innate immune gene expression were performed between the control and field groups using the Mann–Whitney *U* test. A *p*-value less than 0.05 was considered statistically significant.

## Results

### Enhanced performance by droplet digital PCR for detection of *B. dabieense *RNA from *H. longicornis*

**Fig. 1:**
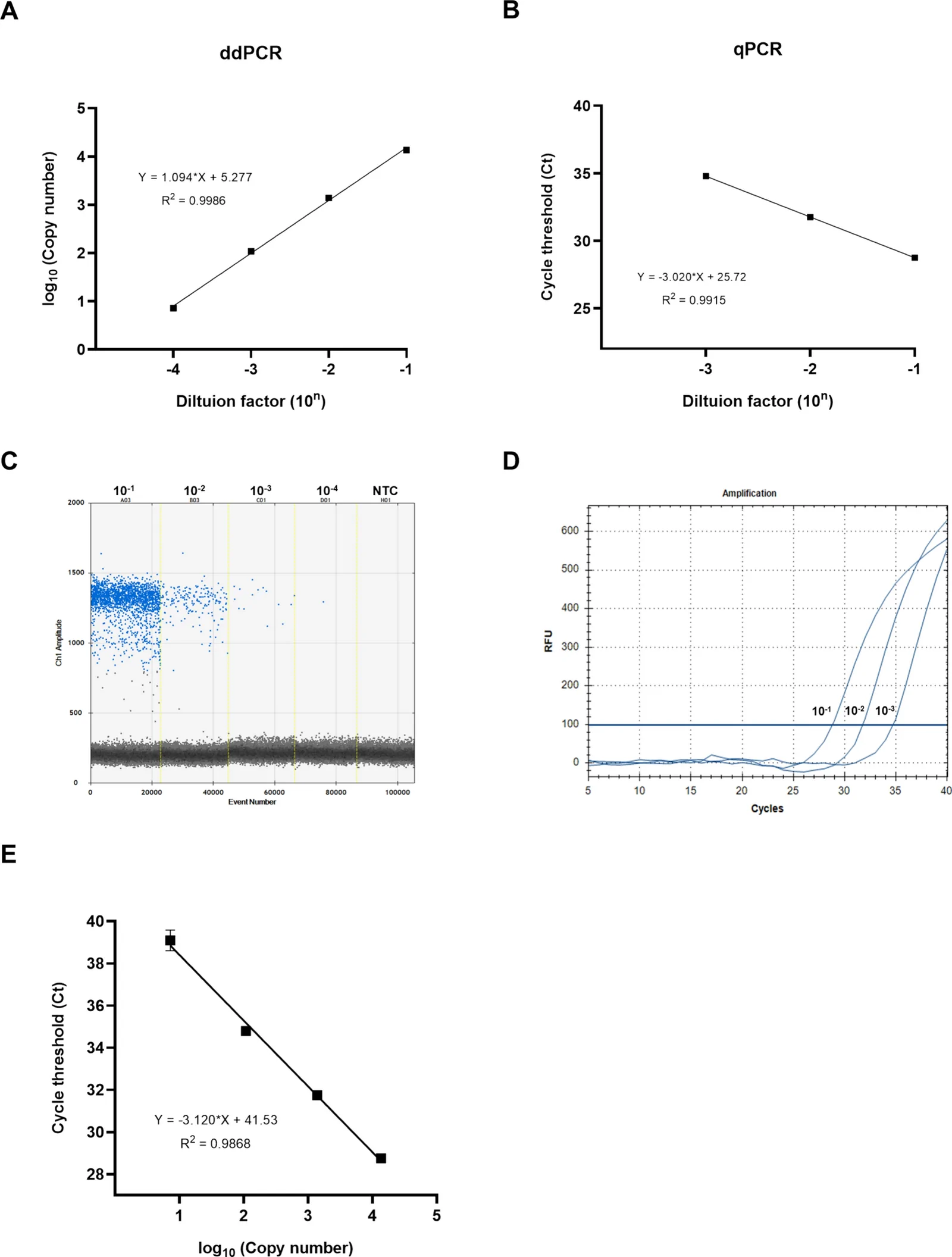
Comparison of detection limits between ddPCR and qPCR for detecting *B. dabieense* RNA. **A** Linear regression of ddPCR was performed using tenfold serial dilutions (10^*n*^) and log₁₀-transformed RNA copy numbers. All experiments were performed in triplicate, and data are presented as mean ± SEM. **B** Standard curve of qPCR generated using tenfold serial dilutions (10*ⁿ*) and corresponding cycle threshold (Ct) values. Data represent the mean ± SEM of triplicate measurements. **C** One-dimensional amplitude plots for ddPCR across the dilution series (10^–1^ to 10^–4^) and no-template control (NTC). Positive droplets (above the fluorescence threshold) are shown in blue and negative droplets are shown in gray. The vertical dashed lines mark the boundaries between each dilution. **D** qPCR amplification curves for the same dilution series. The horizontal line indicates the fluorescence threshold used to determine Ct values, and each curve is labeled according to its dilution factor. **E** Correlation between ddPCR and qPCR measurements for *B. dabieense* RNA detection. The linear regression shows the relationship between log_10_-transformed copy numbers of ddPCR (*x*-axis) and Ct values of qPCR (*y*-axis)

Assay linearity and detection limit of ddPCR and qPCR was directly compared using the identical template *B. dabieense* RNA isolated from the reference material and in-house universal primers and probes (Table 1). Both ddPCR and qPCR showed high linearity between the log10-transformed RNA copy number and dilution factor where *R*^2^ value was 0.9986 and 0.9915, respectively (Fig. 1a, b). The specificity in-house assay was validated by the generation of distinct positive droplet clusters, which was consistently observed in samples across every dilution ranging from 10^–1^ to 10^–4^ (Fig. 1c). *B. dabieense* RNA was detected by ddPCR even at the lowest dilution which was at 10^–4^, and the analytical limit of detection (LoD) for ddPCR was defined as 7.23 copies/µL based on the lowest dilution that consistently yielded positive droplets (Fig. 1a, c, Additional file 1: Fig. S1A). In contrast, for qPCR, the 10^–4^ dilution yielded a Ct value of 39.09, whereas the 10⁻^3^ dilution yielded a Ct value of 34.8 (Fig. 1b, d; Additional file 1: Fig. S1B). The 10^–3^ dilution was defined as the analytical LoD because it represented the lowest dilution that consistently produced amplification across replicate reactions. LoD of qPCR was determined as 144.63 copies/μL by calculating correlation between qPCR Ct values and *B. dabieense* RNA copy numbers determined by ddPCR (Fig. 1e). Our comparison results indicated that ddPCR has at least 20 times higher detection performance than qPCR. Additionally, to evaluate the analytical specificity of the assays in environmental samples, we examined whether tick-derived matrices potentially generate non-specific amplification signals. Based on the amplification curve of qPCR and 1-D amplitude plot of ddPCR, non-specific amplification was not observed in both qPCR and ddPCR assays, confirming that assay specificity was maintained even in the presence of complex tick-derived nucleic acids (Additional file 1: Fig. S2).

**Fig. 2:**
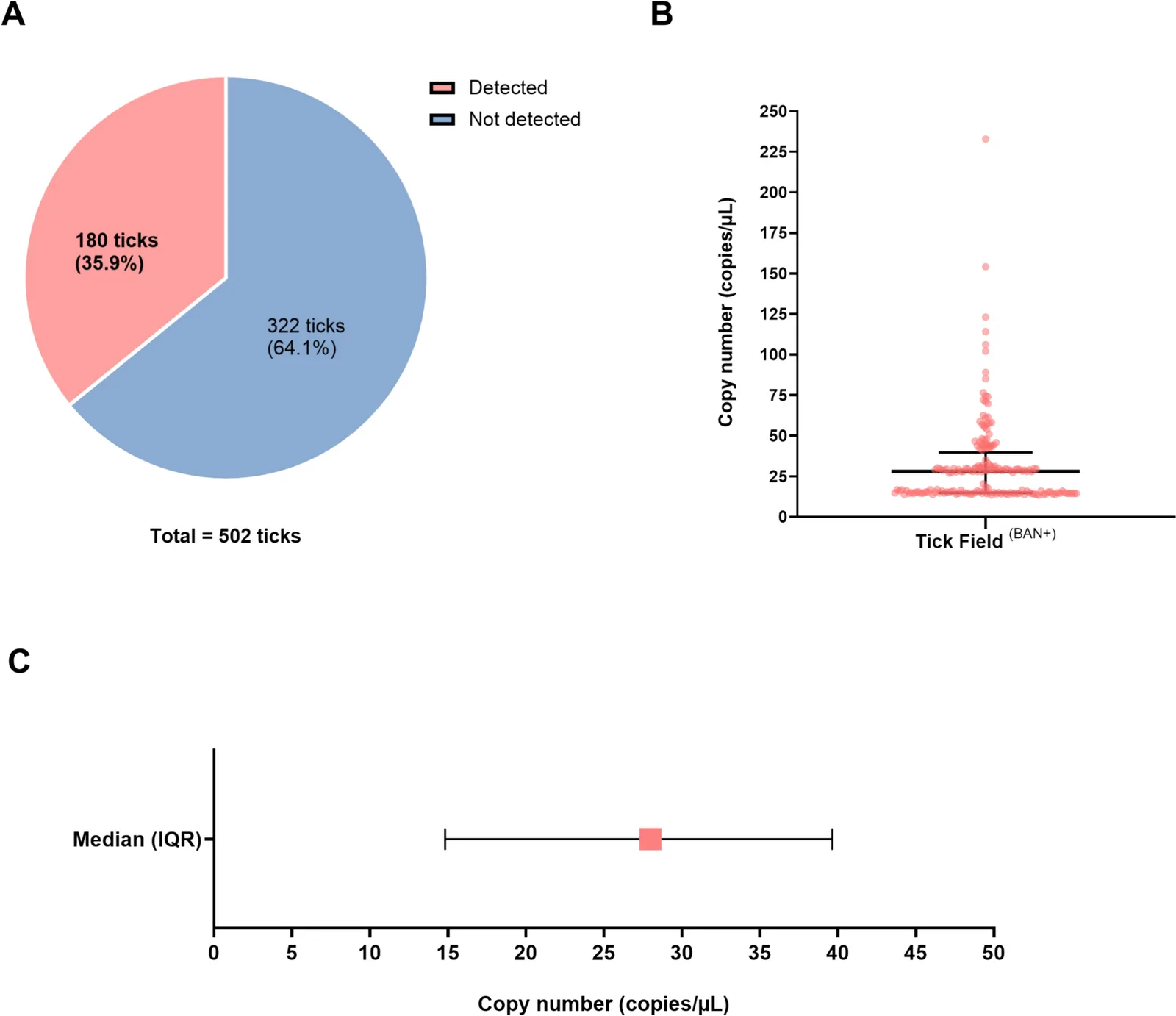
Detection of *B. dabieense* RNA from field-collected *H. longicornis* ticks using ddPCR. **A** Pie chart showing the number of *H. longicornis* ticks (*n* = 502) that were detected (180 ticks, 35.9%) and not detected (322 ticks, 64.1%) for *B. dabieense* by ddPCR. **B** Dot plot showing the distribution of *B. dabieense* RNA copy numbers measured by ddPCR in *H. longicornis* ticks. **C** Median and interquartile range (IQR) of *B. dabieense* RNA copy numbers. The plot displays the median (red square) and the IQR, representing the lower (25%) and upper (75%) quartiles of B. dabieense RNA copy numbers

After ddPCR assay was evaluated using *B. dabieense* RNA reference materials, individual *H. longicornis* ticks were examined for the infection of *B. dabieense*. Among 502 individual *H. longicornis* collected from the field, *B. dabieense* RNA was detected in 180 ticks and the virus detection rate was 35.9% (Fig. 2a). Since ddPCR can directly enumerate target nucleic acid molecules, we examined for *B. dabieense* RNA concentrations from infected individual ticks which ranged from 13.30 to 232.84 copies/µL (Fig. 2b). The median copy number of *B. dabieense* RNA was 27.98 copies/µL (Fig. 2c). The interquartile range (IQR), which indicates the bottom 25% and top 25% of identified individual viral RNA concentrations, was 14.82 and 39.64 copies/µL, respectively (Fig. 2c). Only two individual ticks showed *B. dabieense* RNA copy numbers within the LoD of qPCR method among 180 infected ticks, which indicated that the presence of *B. dabieense* RNA would not be detected accurately in most ticks using qPCR-based approaches (Additional file 1: Table S2).

**Fig. 3:**
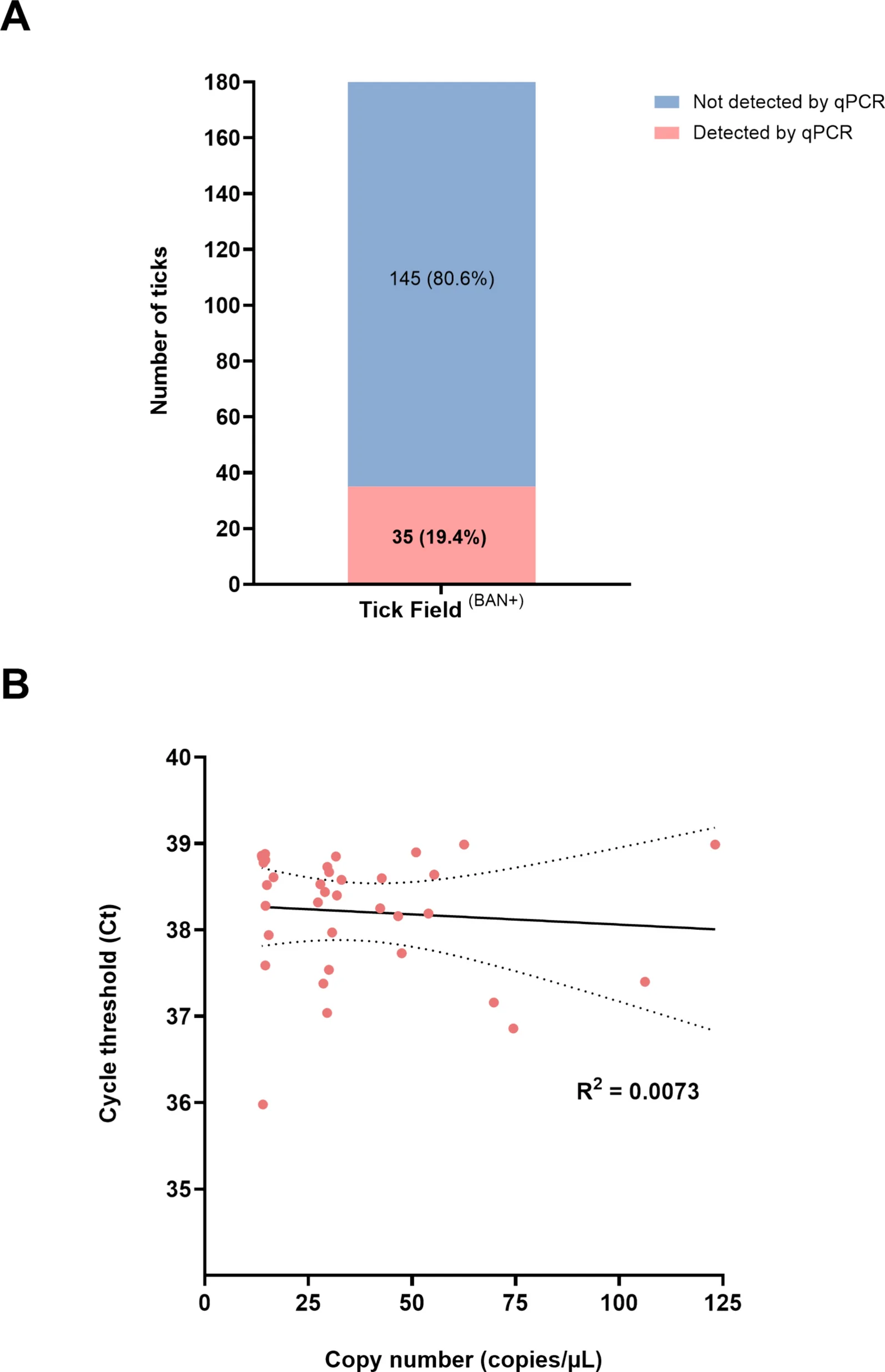
Detection results of *B. dabieense* from ddPCR-positive ticks using qPCR. **A** The stacked bar chart shows the qPCR results of *B. dabieense* detection in *H. longicornis* ticks that were previously confirmed by ddPCR. **B** Correlation between ddPCR and qPCR measurements for *B. dabieense* detection in *H. longicornis* ticks. The scatter plot shows the relationship between copy numbers measured by ddPCR (*x*-axis) and cycle threshold (Ct) values of qPCR (*y*-axis) for *B. dabieense* detection in *H. longicornis* ticks

We also examined the detection rate of qPCR method from the exact samples used for ddPCR analysis. Among 180 ticks previously confirmed by ddPCR, only 35 ticks were detected for the presence of *B. dabieense* RNA by qPCR. This accounts for 19.4% of the detection rate of ddPCR (Fig. 3a). The Ct values for all 35 samples were higher than 34.8 which was outside of the detection range of qPCR (Additional file 1: Table S2). Under these conditions, there was essentially no correlation (*R*^2^ = 0.0073) between copy numbers measured by ddPCR and Ct values of qPCR (Fig. 3b).

### Innate immune response analysis as a complementary indicator for identifying *B. dabieense*-infected *H. longicornis*

When *H. longicornis* ingests pathogens from infected blood meals, effector molecules including antimicrobial peptides, complement-like molecules and reactive oxygen species are produced as tick immune system is activated via immune-signaling pathways [17]. Activated transcription factor within immune-signaling pathways, such as STAT from JAK/STAT pathway, is identified as the main controller for production of effector molecules and pathogen response in ticks [17–19]. In this context, STAT gene expression can serve as a marker for immune activation within ticks in response to viral challenge. Because ddPCR allows absolute quantification of *B. dabieense* RNA from individual ticks, we investigated whether innate immune activation, as reflected by STAT expression, differed between ticks with low and high *B. dabieense* RNA copy numbers. To address this, we analyzed the expression levels of STAT gene from individual ticks infected with less than 14.82 copies/µL of *B. dabieense* RNA, which was the bottom 25% of viral RNA concentrations determined by IQR analysis (Fig. 2c).

**Fig. 4:**
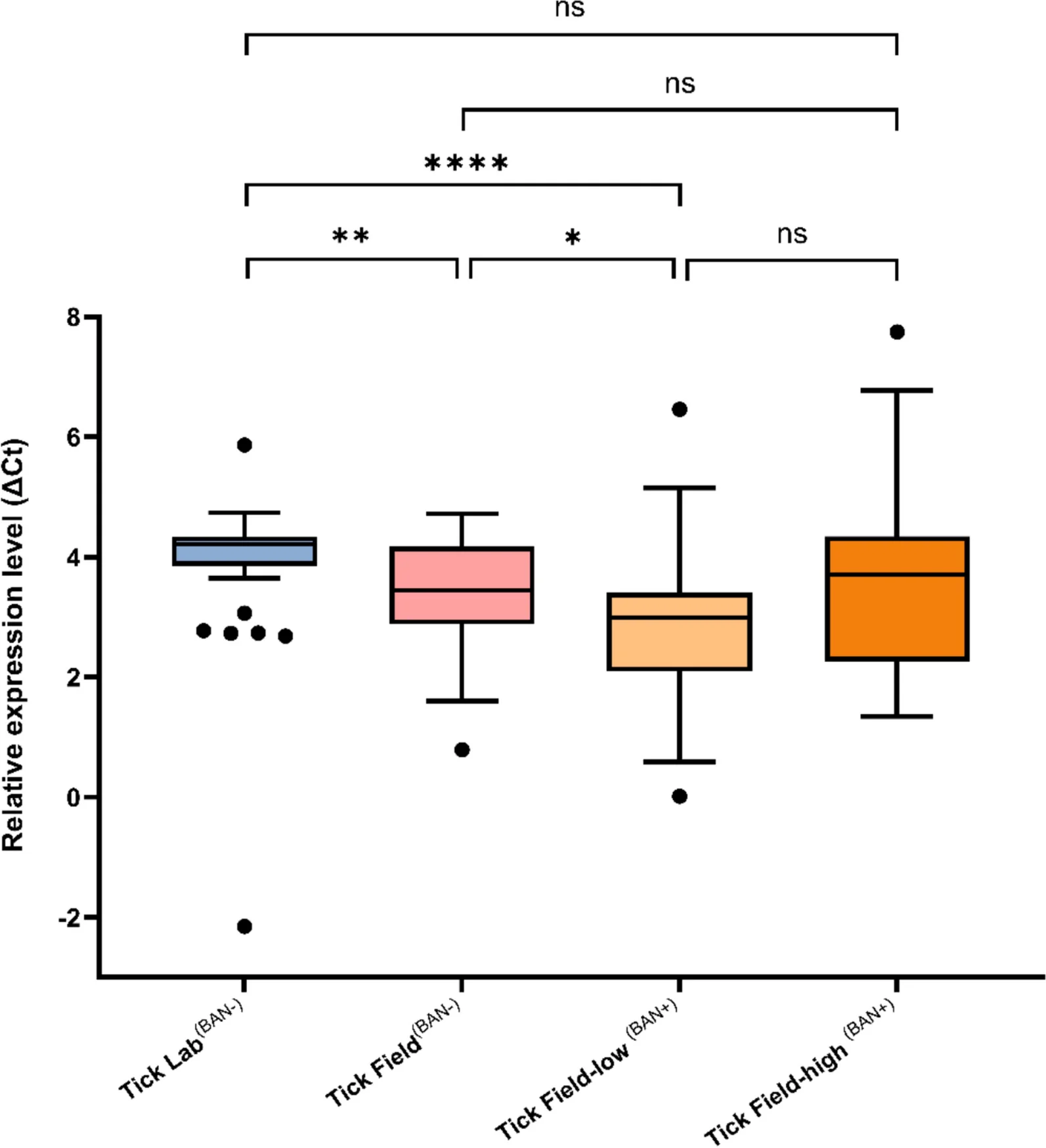
Comparison of STAT gene expression levels from laboratory-reared and field-collected *Haemaphysalis longicornis* ticks. Relative expression levels of STAT, measured by ΔCt values, was compared among four groups: Tick Lab^(BAN−)^ (laboratory-reared, pathogen-negative), Tick Field^(BAN−)^ (field-collected, pathogen-negative), Tick Field-low^(BAN+)^ (field-collected, *B. dabieense*-only detection with low RNA copy numbers, < 15 copies/µL) and Tick Field-high^(BAN+)^ (field-collected, *B. dabieense*-only detection with high RNA copy numbers, > 40 copies/µL). Statistically significant differences are indicated by asterisks: *p* < 0.05 (*), *p* < 0.01 (**), *p* < 0.0001 (****), as determined by appropriate statistical tests. The box plots represent the interquartile range, median and outliers

The relative expression levels, ΔCt, of the *H. longicornis* STAT gene were compared among laboratory-reared ticks (Tick Lab^(BAN−)^), non-infected field-collected ticks (Tick Field^(BAN−)^) and *B. dabieense*-infected field-collected ticks with detected viral RNA below 15 copies/µL (Tick Field-low^(BAN+)^) or above 40 copies/µL (Tick Field-high^(BAN+)^) (Fig. 4). The median ΔCt of Tick Field^(BAN−)^ was 3.447 which was significantly lower than that of Tick Lab^(BAN−)^, 4.223, with the *p*-value of 0.0064 (Fig. 4). Interestingly, the median ΔCt of Tick Field-low^(BAN+)^ was 2.990 which was significantly lower than that of both Tick Field^(BAN−)^ and Tick Lab^(BAN−)^ with *p*-values of 0.0412 and 0.0001, respectively (Fig. 4). The median ΔCt of the Tick Field-high^(BAN+)^ was 3.706, but the differences were not statistically significant compare to other tick populations (Fig. 4).

### Detection of pathogens co-infected with *B. dabieense* from field-collected *H. longicornis*

Universal primers and probes were designed for each tick-borne pathogen by targeting consensus regions of DNA sequences from multiple strains or species (Additional file 1: Table S1 and Additional file 1: Fig. S3). The in-house designed universal primers and probes for each pathogen were validated by ddPCR before the sample analysis (Additional file 1: Figs. S4 and S5). Distinct positive droplet clusters were observed in each universal assay using DNA templates from *O. tsutsugamushi*, *A. phagocytophilum*, *C. burnetii*, *Borrelia* spp. and *Babesia* spp. (Additional file 1: Fig. S4A–E) while no amplification was detected in non-target controls (Additional file 1: Fig. S5A–F). Thus, we confirmed the high specificity of the in-house primer–probe sets for each pathogen.

**Fig. 5:**
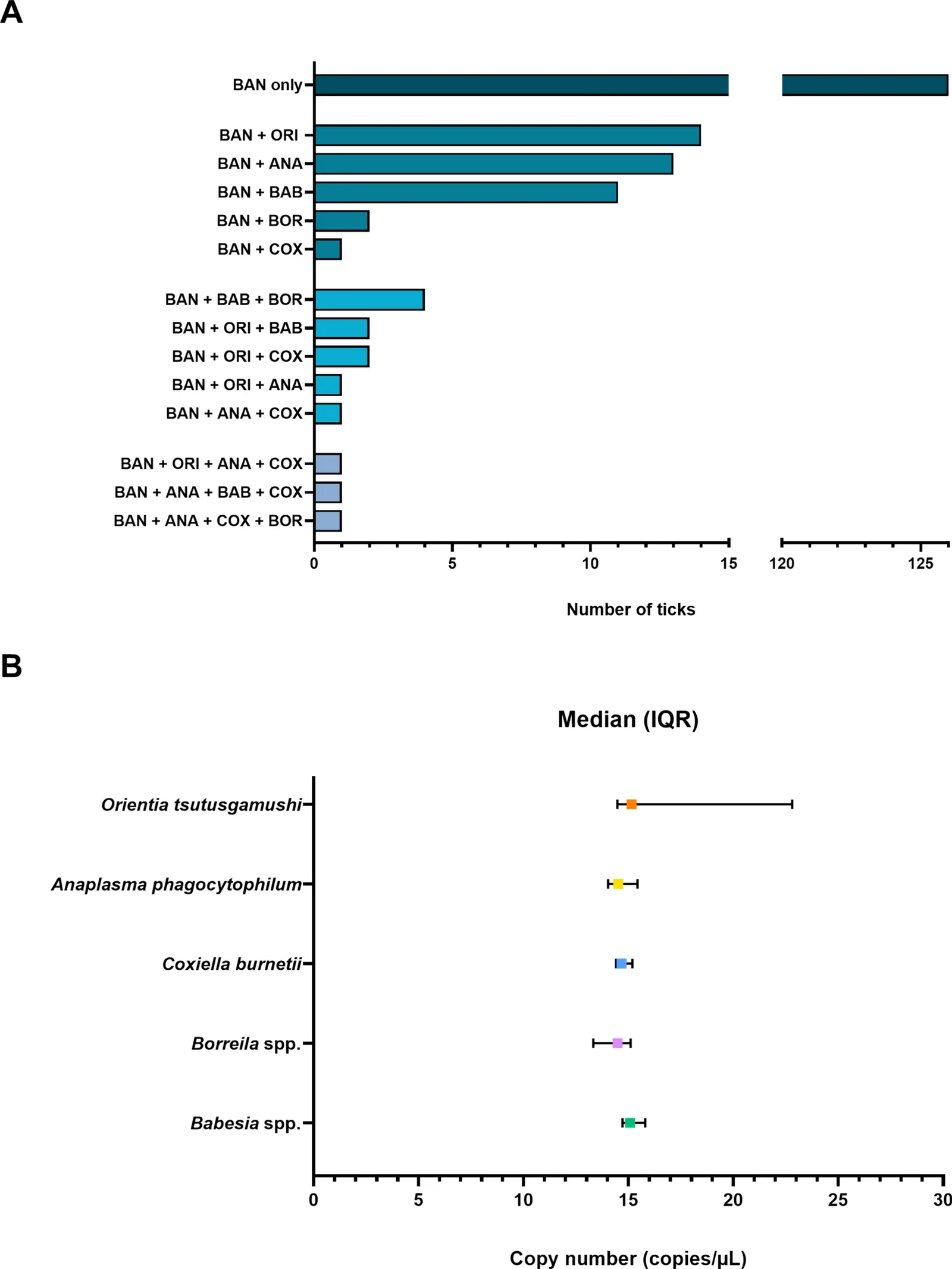
Detection of tick-borne pathogens from *H. longicornis* ticks co-infected with *B. dabieense*. **A** A bar chart showing the number of ticks for each combination of tick-borne pathogens detected by ddPCR, ranging from single to quadruple detections. BAN, *Bandavirus dabieense*; ORI, *Orientia tsutsugamushi*; ANA, *Anaplasma phagocytophilum*; BAB, *Babesia* spp.; COX, *Coxiella burnetii*; BOR, *Borrelia* spp. **B** A plot displaying the median and IQR of copy numbers identified for different tick-borne pathogens. The dots indicate the median and the whiskers represents the IQR

Once we validated the performance of in-house assays, we further examined for the presence of other pathogens in ticks from the previously confirmed *B. dabieense*-infected *H. longicornis* by ddPCR. Among previously identified 180 ticks infected with *B. dabieense*, 126 ticks (70.0%) were infected only with *B. dabieense* while 41 ticks (22.78%) were infected with two pathogens, 10 ticks (5.56%) were infected with three pathogens, and 3 ticks (1.66%) were infected with four pathogens concurrently (Fig. 5a). In summary, our results suggest that most *H. longicornis* ticks from the field were infected with *B. dabieense* alone, which accounted for 70% of identified populations, while 30% of ticks were infected with more than one pathogen at the same time. The major pathogen species co-infected with *B. dabieense* in ticks were *O. tsutsugamushi*, *A. phagocytophilum* and *Babesia *spp. (Fig. 5a).

We also measured pathogen concentrations using the total nucleic acids isolated from infected individual ticks by ddPCR. The median concentration of *O. tsutsugamushi* DNA from infected ticks was 15.15 copies/µL and the IQR was from 14.47 to 22.80 copies/µL (Fig. 5b). The median concentration of *A. phagocytophilum* DNA from infected ticks was 14.51 copies/µL and the IQR was from 14.03 to 15.43 copies/µL (Fig. 5b). The median concentration of *Babesia* spp. DNA from infected ticks was 15.08 copies/µL and the IQR ranged from 14.72 to 15.80 copies/µL (Fig. 5b). The median concentration of *C. burnetii* DNA detected from infected ticks was 14.67 copies/µL and the IQR was 14.40 and 15.19 copies/µL (Fig. 5b). Lastly, the median concentration of *Borrelia* spp. DNA detected from infected ticks was 14.48 copies/µL and the IQR was from 13.33 to 15.11 copies/µL (Fig. 5b).

## Discussion

The ddPCR system is based on partitioning the reaction mixture into tens of thousands of nanoliter-sized droplets, followed by independent amplification of target molecules within each droplet [20, 21]. This partitioning effectively concentrates rare nucleic acid targets into individual reaction compartments and distributes potential PCR inhibitors across droplets, so that at least a subset of partitions can support efficient amplification even in complex matrices such as tick homogenates [22, 23]. In addition, ddPCR relies on binary end-point fluorescence readouts rather than real-time Ct values, which are more sensitive to variation in amplification efficiency, enabling more accurate quantification when efficiency is partially compromised [24, 25]. In this study, we developed a ddPCR-based pathogen monitoring method for the surveillance of *H. longicornis* ticks infected with pathogens in nature. By taking advantage of the technical strengths of ddPCR, we aimed to evaluate whether ddPCR method could enhance the detection of *B. dabieense* and other tick-borne pathogens relative to conventional qPCR-based approaches.

Our ddPCR method showed 20 times lower limit of detection for *B. dabieense* RNA compared to qPCR method, which was 7.23 copies/μL and 144.63 copies/μL, respectively (Fig. 1). This finding is consistent with previous studies comparing the LoD of ddPCR and qPCR for pathogen detection including malaria parasites and other viruses [26, 27]. We observed a detection rate of 35.9% for *B. dabieense*-infected ticks from the field, whereas previous surveillance studies using qPCR have reported prevalence estimates ranging from 0.48 to 12.6% [6–9] (Fig. 2). Although this difference is in accordance with the higher analytical sensitivity of ddPCR observed in this study, it is important to note that variations in geographic location, seasonality and sampling or sample‑handling protocols between studies may also contribute to the observed differences in prevalence [28, 29]. Therefore, our findings suggest that ddPCR can reveal more cases of *B. dabieense* infections at lower concentrations than qPCR, but the absolute prevalence should be interpreted in the context of these methodological and ecological differences.

The median *B. dabieense* RNA concentration from infected *H. longicornis* was 27.98 copies/µL which was outside of the detection limit by qPCR but well within the ddPCR method. Moreover, qPCR-based detection only identified 19.4% of ddPCR‑confirmed ticks with *B. dabieense* infection, and all corresponding Ct values were close to or beyond the qPCR LoD defined in our analytical experiments (Fig. 3). Under these conditions, most *B. dabieense*-positive ticks harbored viral RNA copy numbers near or below the LoD of qPCR. In this low-copy regime, Ct values are expected to show non-linear behavior and high variability, which likely contributes to the essentially absent linear correlation between ddPCR copy numbers and qPCR Ct values (*R*^2^ = 0.0073).

We additionally performed an exploratory analysis of tick innate immune signaling to evaluate whether STAT expression is associated with *B. dabieense* infection near the detection limit of ddPCR. Field-collected *H. longicornis* ticks infected with low concentrations of *B. dabieense* showed statistically higher STAT expression compared with uninfected field populations, whereas ticks with higher viral loads did not differ significantly from other groups (Fig. 4). Given the non-specific nature of STAT as a transcription factor that can be activated by multiple environmental stimuli, these findings should be regarded as hypothesis-generating rather than as evidence for a specific or quantitative surrogate marker of *B. dabieense* infection. Within this framework, our results suggest that immune readouts such as STAT may provide complementary biological context alongside ddPCR-based detection for low-copy infections, but this interpretation remains speculative and will require time-resolved, mechanistic studies to be rigorously tested.

Lastly, we also examined whether field-collected *H. longicornis* were infected with other pathogens concurrently and identified that an individual *H. longicornis* can carry multiple pathogens up to different species at the same time including *B. dabieense*, *O. tsutsugamushi*, *A. phagocytophilum*, *C. burnetii.*, *Borrelia* spp. and *Babesia* spp. (Fig. 5a). In human, several clinical studies reported that molecular and serological diagnosis identified the presence of other tick-borne pathogens from SFTS patients [30, 31]. Notably, co-infection with *B. dabieense* and *O. tsutsugamushi* are observed in high frequency [32–34]. Taking our findings and previous clinical reports together, we confirmed that *H. longicornis* in nature can be infected with multiple pathogens at the same time, and the infected ticks potentially transmit more than one pathogen simultaneously to humans through tick bites. Overall, ddPCR-based quantification indicated that the DNA copy numbers of pathogens detected in co-infected ticks were generally low and fell within a relatively narrow range (Fig. 5b). Given the limited number of co-infected ticks included in the dataset and the restricted spatial and temporal scope of the field sampling, these findings should be interpreted as an exploratory description of co-infection patterns rather than definitive evidence of characteristic infection intensities. In this context, our results show that it is feasible to use multiplex ddPCR to identify combinations of pathogens in naturally infected *H. longicornis* ticks.

## Conclusions

Taken together, our findings indicate that a ddPCR-based pathogen surveillance system can enhance the detection and quantification of *H. longicornis* infected with *B. dabieense* and other tick-borne pathogens compared with qPCR, particularly at low nucleic acid concentrations. Analyses of tick innate immune responses, such as STAT expression, may provide complementary biological context for interpreting low-copy infections identified by ddPCR. In addition, multiplex ddPCR enables simultaneous detection of co-infections with multiple tick-borne pathogens at generally low copy numbers in individual ticks. However, because ddPCR can detect very low RNA levels of uncertain biological relevance, positive results at trace target concentrations should be interpreted cautiously and in conjunction with biological and epidemiological context. Furthermore, since sequencing was not performed on representative amplification products obtained from field samples, it should be noted that the possibility of low-frequency non-target amplification cannot be completely ruled out, particularly for short amplicons at very low copy numbers. Finally, our conclusions are based on a limited number of field samples from one region, so further validation in broader settings will be needed to generalize these findings. Despite these limitations, our results support ddPCR as a promising platform that can be further refined and scaled for integrated surveillance and management of tick-borne zoonoses in vector populations.

## Supplementary Information

Additional file 1.

## Data Availability

All pertinent data for the study are either presented in the article or available as supplementary information.
