# Supplementary material for: Droplet digital PCR-based pathogen monitoring system improves identifying Haemaphysalis longicornis infected with Bandavirus dabieense and other tick-borne pathogens at low concentrations
Source: Parasit Vectors. 2026 Jul 1;19:400. doi: 10.1186/s13071-026-07548-1 (PMC13595599; doi:10.1186/s13071-026-07548-1)
Supplement: Supplementary file 1 [file 13071_2026_7548_MOESM1_ESM.docx]

**Supplementary data**

**Droplet digital PCR-based pathogen monitoring system improves identifying *Haemaphysalis longicornis* infected with *Bandavirus dabieense* and other tick-borne pathogens at low concentrations**

Yujin Baek^1,2^, Jae-Hun Jeong^1,3^, Da-Eun Jeong^4^, Jun-Gu Kang^4^, Yong Shin^1*^, Il-Hwan Kim^1,5**^

^1^Division of Biomedical Metrology, Korea Research Institute of Standards and Science, Daejeon, Republic of Korea

^2^Department of Biotechnology, College of Life Science and Biotechnology, Yonsei University, Seoul, Republic of Korea

^3^Department of Precision Engineering Science, University of Science and Technology, Daejeon, Republic of Korea

^4^Korea Zoonosis Research Institute, Jeonbuk National University, Iksan, Republic of Korea

^5^Department of Precision Measurement Science, University of Science and Technology, Daejeon, Republic of Korea

*Corresponding author: Yong Shin, PhD: [shinyongno1@yonsei.ac.kr](mailto:shinyongno1@yonsei.ac.kr)

**Corresponding author: Il-Hwan Kim, PhD: [ihkim@kriss.re.kr](mailto:ihkim@kriss.re.kr) **Table S1.** Pathogen genotypes, strains and species considered for universal detection in primer and probe design

| **Pathogen** | **Target gene** | **Genotypes / Strains / Species** |
| --- | --- | --- |
| *Badavirus dabieense* | RdRp | Genotype A, B, C, D, E, F |
| *Orientia tsutsugamushi* | 56-kDa | Boryong, Oishi, Kanda |
| *Anaplasma phagocytophilum* | ankA | str. JM |
| *Coxiella burnetii* | IS1111 | RSA 439 (Nine mile), RSA 493, RSA 331 |
| *Borrelia* *spp*. | 16s rRNA | *B. afzelii*, *B. garinii*, *B. valaisiana*, *B. miyamotoi* |
| *Babesia* *spp*. | 18s rRNA | *B. microti*, *B. capreoli*, *B. gibsoni*, *B. venatorum*,  *B. duncani* |

**Table S2.** Quantitative values of *Bandavirus dabieense* RNA from virus-infected *Haemaphysalis longicornis* ticks determined by ddPCR and qPCR.

| **Tick no.^*^** | **Copy number** | **Ct** | **Tick no.** | **Copy number** | **Ct** |  |
| --- | --- | --- | --- | --- | --- | --- |
| **1** | 232.84 | - | **46** | 35.03 | - |  |
| **2** | 154.15 | - | **47** | 32.96 | 38.58 |  |
| **3** | 123.16 | 38.99 | **48** | 31.89 | 38.40 |  |
| **4** | 114.20 | - | **49** | 31.64 | 38.85 |  |
| **5** | 106.20 | 37.40 | **50** | 31.44 | - |  |
| **6** | 102.10 | - | **51** | 31.35 | - |  |
| **7** | 89.06 | - | **52** | 31.15 | - |  |
| **8** | 85.10 | - | **53** | 30.71 | 37.97 |  |
| **9** | 76.54 | - | **54** | 30.69 | - |  |
| **10** | 74.48 | 36.86 | **55** | 30.41 | - |  |
| **11** | 73.99 | - | **56** | 30.18 | - |  |
| **12** | 72.04 | - | **57** | 30.02 | 38.67 |  |
| **13** | 70.97 | - | **58** | 29.93 | 37.54 |  |
| **14** | 69.76 | 37.16 | **59** | 29.92 | - |  |
| **15** | 62.56 | 38.99 | **60** | 29.91 | - |  |
| **16** | 61.47 | - | **61** | 29.87 | - |  |
| **17** | 61.14 | - | **62** | 29.84 | - |  |
| **18** | 58.77 | - | **63** | 29.68 | - |  |
| **19** | 58.15 | - | **64** | 29.64 | - |  |
| **20** | 57.63 | - | **65** | 29.64 | - |  |
| **21** | 57.58 | - | **66** | 29.56 | 38.73 |  |
| **22** | 57.01 | - | **67** | 29.51 | 37.04 |  |
| **23** | 56.21 | - | **68** | 29.49 | - |  |
| **24** | 55.37 | 38.64 | **69** | 29.39 | - |  |
| **25** | 53.97 | 38.19 | **70** | 29.19 | - |  |
| **26** | 50.98 | 38.90 | **71** | 29.18 | - |  |
| **27** | 48.24 | - | **72** | 29.15 | - |  |
| **28** | 47.85 | - | **73** | 29.06 | - |  |
| **29** | 47.51 | 37.73 | **74** | 29.06 | - |  |
| **30** | 46.65 | 38.16 | **75** | 28.98 | - |  |
| **31** | 46.14 | - | **76** | 28.97 | 38.44 |  |
| **32** | 45.74 | - | **77** | 28.97 | - |  |
| **33** | 44.97 | - | **78** | 28.89 | - |  |
| **34** | 44.55 | - | **79** | 28.80 | - |  |
| **35** | 44.29 | - | **80** | 28.80 | - |  |
| **36** | 44.27 | - | **81** | 28.71 | - |  |
| **37** | 44.09 | - | **82** | 28.67 | - |  |
| **38** | 44.05 | - | **83** | 28.67 | - |  |
| **39** | 43.75 | - | **84** | 28.64 | - |  |
| **40** | 43.17 | - | **85** | 28.59 | 37.38 |  |
| **41** | 42.96 | - | **86** | 28.49 | - |  |
| **42** | 42.69 | 38.60 | **87** | 28.41 | - |  |
| **43** | 42.33 | 38.25 | **88** | 28.28 | - |  |
| **44** | 42.19 | - | **89** | 28.01 | - |  |
| **45** | 41.18 | - | **90** | 28.00 | - |  |
| **Tick no.** | **Copy number** | **Ct** | **Tick no.** | **Copy number** | **Ct** | |
| **91** | 27.96 | - | **136** | 14.82 | - | |
| **92** | 27.93 | - | **137** | 14.79 | - | |
| **93** | 27.88 | 38.53 | **138** | 14.77 | - | |
| **94** | 27.87 | - | **139** | 14.75 | - | |
| **95** | 27.78 | - | **140** | 14.75 | - | |
| **96** | 27.74 | - | **141** | 14.71 | - | |
| **97** | 27.32 | 38.32 | **142** | 14.63 | 38.28 | |
| **98** | 27.32 | - | **143** | 14.62 | 37.59 | |
| **99** | 26.90 | - | **144** | 14.61 | 38.81 | |
| **100** | 20.34 | - | **145** | 14.60 | - | |
| **101** | 18.12 | - | **146** | 14.59 | - | |
| **102** | 17.71 | - | **147** | 14.58 | - | |
| **103** | 16.91 | - | **148** | 14.57 | 38.88 | |
| **104** | 16.70 | - | **149** | 14.55 | - | |
| **105** | 16.69 | - | **150** | 14.49 | - | |
| **106** | 16.56 | 38.61 | **151** | 14.46 | - | |
| **107** | 16.53 | - | **152** | 14.45 | - | |
| **108** | 16.40 | - | **153** | 14.43 | - | |
| **109** | 16.09 | - | **154** | 14.43 | - | |
| **110** | 15.97 | - | **155** | 14.40 | - | |
| **111** | 15.95 | - | **156** | 14.39 | - | |
| **112** | 15.93 | - | **157** | 14.37 | - | |
| **113** | 15.93 | - | **158** | 14.35 | - | |
| **114** | 15.88 | - | **159** | 14.34 | - | |
| **115** | 15.86 | - | **160** | 14.30 | - | |
| **116** | 15.82 | - | **161** | 14.28 | - | |
| **117** | 15.81 | - | **162** | 14.20 | - | |
| **118** | 15.63 | - | **163** | 14.18 | - | |
| **119** | 15.53 | - | **164** | 14.16 | 38.78 | |
| **120** | 15.47 | - | **165** | 14.15 | - | |
| **121** | 15.44 | 37.94 | **166** | 14.13 | - | |
| **122** | 15.32 | - | **167** | 14.11 | - | |
| **123** | 15.27 | - | **168** | 14.05 | - | |
| **124** | 15.27 | - | **169** | 14.01 | 35.98 | |
| **125** | 15.25 | - | **170** | 13.99 | - | |
| **126** | 15.19 | - | **171** | 13.94 | - | |
| **127** | 15.16 | - | **172** | 13.86 | - | |
| **128** | 15.06 | - | **173** | 13.84 | 38.84 | |
| **129** | 15.00 | 38.52 | **174** | 13.77 | - | |
| **130** | 14.97 | - | **175** | 13.74 | 38.86 | |
| **131** | 14.95 | - | **176** | 13.74 | - | |
| **132** | 14.90 | - | **177** | 13.65 | - | |
| **133** | 14.89 | - | **178** | 13.64 | - | |
| **134** | 14.87 | - | **179** | 13.37 | - | |
| **135** | 14.82 | - | **180** | 13.30 | - | |

^*^Unique identification number assigned to each *H. longicornis* tick samples arranged by descending order based on the *B. dabieense* RNA copy number.

**Figure S1.** Determination of analytical limits of detection (LoD) for ddPCR and qPCR. The analytical LoD was defined as the lowest RNA concentration that yielded positive results in all three replicate reactions, with consistent positive droplets or amplification curves within the quantitative range of each assay. (A) One‑dimensional ddPCR amplitude plot for *B. dabieense* RNA at 10⁻³ and 10⁻⁴ dilution factors. (B) qPCR amplification curves for *B. dabieense* RNA at 10⁻³ and 10⁻⁴ dilution factors.

**Figure S2.** Evaluation of analytical specificity of the ddPCR and qPCR assays in environmental tick samples. (A) qPCR amplification curves obtained from three representative tick homogenate samples with and without spiking of *B. dabieense* reference material. (B) One-dimensional ddPCR amplitude plots for the same tick homogenate samples with and without spiking of *B. dabieense* reference material.

**Figure S3.** Multiple sequence alignments of target gene regions for universal primer and probe design**.** Sequence alignments of representative genotypes, strains or species for each tick-borne pathogen. (A) Sequence alignment of *B. dabieense* genotypes. (B) Sequence alignment of *O. tsutsugamushi* strains. (C) Sequence alignment of *C. burnetii* strains. (D) Sequence alignment of *Borrelia* species. (E) Sequence alignment of *Babesia* species. The forward and reverse primers, along with the probe sequences, are highlighted in red.

**Figure S4**. Validation of ddPCR assays for tick-borne pathogen detection. (A) One-dimensional ddPCR amplitude plot of *O.* *tsutsugamushi*. (B) One-dimensional ddPCR amplitude plot of *A. phagocytophilum*. (C) One-dimensional ddPCR amplitude plot of *C. burnetti*. (D) One-dimensional ddPCR amplitude plot of *Borrelia spp*. (E) One-dimensional ddPCR amplitude plot of *Babesia spp*. Positive droplets are shown in green (HEX) or blue (FAM), and negative droplets are displayed in gray. PC, positive control; NTC, no-template control.

**Figure S5.** ddPCR 1D-plots for the non-target control of universal assays for each pathogen. The ddPCR 1D-plots show the results of the assays with non-target control groups, where no amplification was observed in all targets. (A) One-dimensional ddPCR amplitude plot of *B. dabieense*. (B) One-dimensional ddPCR amplitude plot of *O.* *tsutsugamushi*. (C) One-dimensional ddPCR amplitude plot of *A. phagocytophilum*. (D) One-dimensional ddPCR amplitude plot of *C. burnetti*. (E) One-dimensional ddPCR amplitude plot of *Borrelia spp*. (F) One-dimensional ddPCR amplitude plot of *Babesia spp*. NTC, no template control.
